# Community service rehabilitation therapists’ perspectives of cross-disciplinary supervision

**DOI:** 10.4102/safp.v67i1.6077

**Published:** 2025-02-21

**Authors:** Zinzile N. Sibiya, Andrew J. Ross

**Affiliations:** 1 Department of Family Medicine, College of Health Sciences, University of KwaZulu-Natal, Durban, South Africa

**Keywords:** supervision, community service, support, rehabilitation therapist, KwaZulu-Natal province

## Abstract

**Background:**

In South Africa, graduate rehabilitation therapists undertake a compulsory community service (CS) year in public healthcare facilities, often entering this role without sufficient competency to work independently. They rely on supervision and support, which may come from senior therapists of different disciplines. This study, conducted in KwaZulu-Natal (KZN) province, explores the experiences of rehabilitation therapists regarding cross-disciplinary supervision and support during their CS year.

**Methods:**

A qualitative, descriptive, and exploratory design was employed. Virtual semi-structured interviews were conducted with seven purposively sampled participants from five KZN districts. Data were audio recorded, transcribed verbatim, and analysed thematically.

**Results:**

The findings revealed four themes and ten sub-themes, highlighting the challenges and benefits of cross-disciplinary supervision, dissatisfaction with existing support structures, and recommendations for improvement. The CS therapists, as junior staff, often lacked the experience and authority to deliver optimal patient care within resource-constrained settings.

**Conclusion:**

While discipline-specific supervision remains the preferred approach, cross-disciplinary supervision contributed positively to professional development, broadening therapists’ understanding of other rehabilitation disciplines. However, the limited availability of accessible, discipline-specific supervisors remains a significant concern. Given their frequent isolation and minimal experience, CS therapists require consistent, discipline-specific support to meet service demands.

**Contribution:**

This study emphasises need to address the experiences of CS therapists, who face high patient loads and limited support, in public health sector planning. Incorporating their insights can enhance their ability to deliver essential services, ensuring better outcomes for the populations served by the public health system.

## Introduction

In South Africa (SA), graduate rehabilitation therapists (speech therapists, occupational therapists, physio therapists and audiologists) are required to complete a compulsory year of community service (CS) in public healthcare facilities. This initiative aims to ensure adequate provision of essential rehabilitation services in underserved communities. Despite being recent graduates, these therapists are not yet considered fully competent to work independently, and thus require supervision and support from senior rehabilitation therapists. However, this support is often lacking, especially in rural areas where the shortage of qualified therapists poses significant challenges.

The CS year serves as an opportunity for young professionals to develop critical thinking skills, increase their knowledge and experience base, and enhance their professional growth. Since CS is promoted as providing ‘services’ rather than training, the primary focus has been on meeting community needs rather than ensuring adequate supervision and support for newly qualified therapists.^1^ The *Health Professions Amendment Act No. 56 of 1998*,^2^ which governs this aspect of their training, emphasises the importance of supervision during CS to ensure therapists are mentored by experienced professionals.^3^ The level of supervision available significantly impacts the functioning of these newly qualified healthcare professionals (HCPs) and their ability to deliver high-quality medical treatment.^1^

Ideally, according to the National Department of Health, rehabilitation staff should be supervised by someone from the same discipline, as this enhances professional development during CS. However, because of a shortage of professional staff, new rehabilitation graduates are often supervised by HCPs from other disciplines, primarily from the rehabilitation field; although in some cases, supervision may extend to non-rehabilitation professionals when no discipline specific supervisors are available.^4^ While not ideal, cross-disciplinary supervision in public health settings is provided to ensure support and oversight to recent graduates in the absence of suitably qualified personnel. This approach, while not without challenges, offers some level of support and supervision, may promote collaboration across professional fields and can encourage a comprehensive problem-solving approach.^5^ It also has the potential to assist new graduates in gaining a deeper understanding of complex public health issues that can be addressed by collaborating with specialists from various disciplines.^5^

Cross-disciplinary supervision has the potential to be very positive when the diversity of perspectives and skills is acknowledged and valued. It can foster a collaborative approach to problem-solving and decision making, bridging gaps by bringing together supervisors and supervisees from different areas of expertise. This integration of varied viewpoints can lead to creative solutions and high-quality research.^5^ When practitioners from different disciplines work collaboratively to assess and address all parts of a project or process, taking into consideration each discipline’s distinct ideas and viewpoints, optimal results may be achieved.^5^

However, the benefits of diverse viewpoints can be offset by challenges such as miscommunication or a lack of understanding when the supervisors lack specialised knowledge in the supervisee’s field.^6^ Supervisors may struggle to understand the complexity, difficulties or optimal methods associated with the supervisee’s tasks, potentially affecting the quality of supervision. These challenges can also create communication barriers within the team.^7^ In addition, power dynamics may influence cross-disciplinary supervision, with specific disciplines or individuals exerting control, potentially limiting diverse viewpoints or leading to inappropriate decisions.^7,8,9^ The power dynamics in cross-disciplinary supervision are diverse and complex, being influenced by factors such as governance structures, interpersonal relationships and individual characteristics.^8^ Understanding these dynamics is essential for fostering effective supervisory relationships and promoting professional development.

In the health science disciplines of audiology, physiotherapy, speech-language therapy and occupational therapy, new graduates often undertake CS in hospitals and health facilities where they may be the only person in their respective departments, particularly in rural areas, where retention of senior staff is difficult, or the posts are not available. This situation results in the new graduates being supervised either remotely by someone in the same discipline or by a professional in another discipline who may be located at the hospital. Although cross- supervision is increasingly common in health sciences education and practice, there is limited understanding of the issues involved when young graduates are supervised by professionals from different fields.^10^ This lack of information hinders the development of effective cross-supervision models, which could impact the quality of interprofessional education, clinical skills development, professional and personal confidence, and patient care.^10^ This research study investigates how young rehabilitation graduates perceive and navigate cross-supervision, focussing on its impact on their learning, skills development and future professional growth. The study aimed to explore their experiences of being supervised by professionals from other disciplines to enable a better understanding of these dynamics and support the development of guidelines for improving CS supervision.

### Contribution to the field

This study contributes to the understanding of cross-disciplinary supervision by exploring the experiences of rehabilitation therapists during their CS. It highlights the benefits, challenges, opportunities and implications of cross-disciplinary supervision, offering insights to inform and enhance supervisory models. The findings of this study can help relevant stakeholders understand the obstacles faced by new graduates, ultimately supporting improved supervisory practices in resource limited settings.

## Research methods and design

This exploratory, qualitative, descriptive study was conducted virtually between April and June 2024 via WhatsApp calls with seven rehabilitation therapists in their respective settings who were working in 5 out of the 11 health districts in KwaZulu-Natal (KZN) province. All participants worked as CS therapists in KZN public sector district hospitals between 2022 and 2024 and provided out-patient services at clinics, their patients having been referred from primary healthcare facilities.

The study included rehabilitation therapists – physiotherapists, occupational therapists, audiologists and speech-language pathologists – who were either currently completing or had recently completed their mandatory 1-year CS in KZN, South Africa. Participants were required to have been placed in healthcare facilities located in rural or underserved areas of the province to reflect the challenges and dynamics of these settings. Only those who voluntarily consented to participate and were willing to share detailed accounts of their supervision experiences were included. Furthermore, accessibility via the social media platforms used for recruitment was necessary for participation, which influenced the sample selection. These inclusion criteria ensured that the study captured the targeted population’s experiences with cross-disciplinary supervision.

A purposive sampling technique was used by circulating a participant recruitment poster on various social media platforms, such as Facebook, WhatsApp and Instagram. Those interested in participating in the study were requested to contact the principal researcher directly on the number provided. Rehabilitation therapists who met the inclusive criteria were given information sheets with consent forms to complete and sign before the interviews. While recruitment was conducted through social media platforms, additional measures included requesting proof of Health Professions Council of South Africa (HPCSA) registration or CS assignment letters to verify participants’ credentials formally. Seven participants were virtually interviewed using a semi-structured interview guide to collect the data about their experiences. The questions related to their experiences of being cross-disciplinary supervised during their CS year, the recorded interviews lasted for approximately 40 min.

The following questions and prompts guided the interviews:

1. What does cross-disciplinary supervision mean to you?
2. How was your experience of being supervised by someone from a different profession?
  - What was good or not so good?
  - Tell me a story that helps illustrate this.
3. What are your views on cross-disciplinary supervision?
  - Is it a necessity or not? How so?
4. Do you think it benefited you?
  - Yes or No, and How?
5. Do you think having a supervisor within your field would have produced similar outcomes?
6. To what extent do you think being supervised by a professional not in your field affected the way you delivered services?
7. What advice would you give to CS therapists who are being supervised by professionals from a different discipline?
8. How has cross-supervision affected you post CS?

The thematic data analysis followed the guidelines outlined by Braun and Clarke,^11^ which consisted of multiple stages.After reading and re-reading the transcripts, important phrases, ideas and recurring patterns in the participants’ stories were identified, and codes, categories and themes reflecting their experiences were identified. Every theme was defined and assigned a descriptive name that captured its central message, the last step blending the concepts into a cohesive story that emphasised the main results of the research.^12^

Guba’s and Lincoln’s trustworthiness framework was applied, which entailed addressing issues of credibility, dependability, confirmability, transferability and authenticity. The intention was to improve the study’s credibility and rigour by using a framework that focusses on the distinct experiences. Credibility was ensured by using audio-recorded interviews and verbatim transcriptions, with an independent qualitative coder with a rehabilitation professional background and extensive experience in qualitative research also conducting the thematic analysis, and the discrepancies being discussed until consensus was reached. Dependability was ensured by the researcher carefully documenting all research activities, such as data collection techniques and analysis procedures. Confirmability was established by using direct quotes from the participants in the research article and report. Thick descriptions of the study setting and detailing the participant demographics ensured the transferability of the study’s findings to other CS therapists.

When warranting authenticity, the researcher focussed on developing trustworthy connections with the participants, making sure they were at ease when sharing their experiences. Participants were also urged to recount their experiences in their own manner, creating a space where they felt appreciated and esteemed. They were given all the necessary information and confidentiality was ensured throughout the research to enable a more authentic portrayal of their experiences.

### Ethical considerations

Ethical approval was obtained from the University of KZN’s Humanities and Social Sciences Ethics Committee (HSSREC/00006667/2024). Participation was voluntary and participants were informed about of their ability to exit the study at any time. Before the interviews started, all participants verbally gave their consent, which included allowing audio recording. Ethical standards, such as non-maleficence, confidentiality, privacy, anonymity and justice, were maintained, and participants employment locations were kept anonymous.

## Results

Seven semi-structured interviews were conducted with one physiotherapist, three speech and language therapists, one occupational therapist and two audiologists (Table 1). Of the seven participants interviewed, four had supervisors based in the same facility, which allowed for easier access to direct supervision when needed. This was partly influenced by the location of their facilities, as six of the participants were providing services in rural areas. In these rural settings, participants were often the sole therapist in their department, further highlighting the challenges of receiving consistent and discipline specific supervision. While rural placement increased the likelihood of working independently, the availability of on-site supervisors varied, demonstrating that supervision challenges were not solely tied to geographic location.

**TABLE 1:** Participants’ demographic details.

Code	Age	Gender	Discipline	Supervisor discipline	Supervisor location	Number in department	Rural or Urban
P1	24	Female	OT	PT	Onsite accessible	1	Rural
P2	25	Female	SLT	PT	Offsite accessible	1	Rural
P3	23	Female	AUD	SLT	Offsite inaccessible	1	Urban
P4	24	Female	AUD	PT	Onsite accessible	1	Rural
P5	24	Female	PT	AUD	Onsite accessible	1	Rural
P6	25	Female	SLT	PT	Onsite accessible	1	Rural
P7	24	Female	SLT	AUD	Onsite accessible	1	Rural

AUD, audiologist; SLT, speech-language therapist; OT, occupational therapist; PT, physiotherapist.

All the respondents replied to all the questions, with four themes emerging, being further divided into various sub-themes (Table 2).

**TABLE 2:** Themes and sub-themes.

Themes		Sub-theme
1.	Challenges of cross- disciplinary supervision	1.1 The lack of knowledge 1.2 Power dynamics 1.3 Resource constraints
2.	Benefits of cross-disciplinary supervision	2.1 Enhanced learning opportunities 2.2 Learning administrative and management skills 2.3 Holistic patient care
3.	Dissatisfaction with supervision	3.1 The lack of supervision 3.2 Effects on career advancements
4.	Recommendations for adequate cross-disciplinary supervision	4.1 Building collaborative relationships 4.2 Effective communication strategies

### Theme 1: Challenges of cross-disciplinary supervision

Participants shared challenges encountered during their CS year that were associated with the supervision received, which had an impact on the services they rendered and affected their development as novice rehabilitation therapists. Three sub-themes emerged, these being the lack of knowledge, power dynamics and resource constrains.

#### Sub-theme 1.1: The lack of knowledge

Participants highlighted that their supervisors lacked relevant knowledge essential for clinical development in their domain, which led to poor insight into patient management, as they needed a common understanding of certain situations:

‘My supervisor knew nothing about speech therapy … I did not have anyone to ask advice from when cases were difficult for me … Our conversations were not effective.’ (P6, Female, SLT, 25)

#### Sub-theme 1.2: Power dynamics

Power dynamics in cross-disciplinary supervision were reported to be different from those in traditional supervision situations. Within the hospital, and even within the rehabilitation field, some therapists are considered ‘more valued’ than others, which leads to dominance by one discipline or supervisor and affects the service delivery of the other professionals. This was complicated by the difficulty of new graduates expressing their opinions about their profession to a more senior member who was not familiar with that domain:

‘I wish I could advocate for speech therapy just like the disciplines with experienced professionals … My opinions and inputs were not taken seriously because I did not know much, and there was no one to advocate for me.’ (P3, Female, AUD, 23)

#### Sub-theme 1.3: Resource constraints

Resource constraints are a common issue in the public service, the participants noting the difficulties of working in such environments, mainly when their supervisor was unaware of what resources were needed to manage patients. This forced them to refer patients to other institutions where resources were available and reduced the opportunities for CS rehabilitation therapists to gain experience:

‘It was so hard having to see patients with nothing in the department. I had nothing to use for any assessment and therapy.’ (P8, Female, SLT, 23)

It was also mentioned that not having a supervisor knowledgeable about the discipline was challenging, especially when they had to order resources for the department, which often resulted in delayed ordering, or their needs being ignored:

‘At one point, I had to ask a therapist from a nearby hospital to help because it was hard [*to know what to order*], and if I had a SLT supervisor, they would have ordered [*that equipment or those things*] a long time ago, and the department would have the required resources.’ (P7, Female, SLT, 24)

### Theme 2: Benefits of cross-disciplinary supervision

Despite the challenges, some participants expressed positive experiences gained from supervision during their CS year, this being reflected in the three sub-themes of enhanced learning opportunities, learning administrative and management skills as well as holistic patient care.

#### Sub-theme 2.1: Enhanced learning opportunities

Participants noted that not having a supervisor in the same profession encouraged them to do more research on specific management procedures and learn at every opportunity:

‘This experience helped me so much … it forced me to research and be on par with recent practices. I can take pride in what I learned even during difficult times.’ (P1, Female, OT, 24)

#### Sub-theme 2.2: Learning administrative and management skills

Participants found that exposure to cross disciplinary supervision assisted them in developing skills in their leadership approaches and gives them an opportunity to experience growth in managing team dynamics and resolving conflicts. This was often because of them being the only member of their profession in the facility and having to work in a multidisciplinary management approach:

‘I learned how to mediate differences within the team by applying techniques I observed from my supervisor’s management style.’ (P5, Female, PT, 24)

‘Having a supervisor from another discipline forced me to think outside my usual framework. I learned to adjust my approach based on different perspectives … I also believe that the development of my administrative skills will be a bonus post community service.’ (P3, Female, AUD, 23)

‘Working with a supervisor from a different background taught me how to approach challenges from various angles. I learned to adapt my communication style and manage team dynamics more effectively.’ (P8, Female, SLT, 23)

#### Sub-theme 2.3: Holistic patient care

Participants reported that during this experience, they gained a more in-depth understanding of holistic patient care. Therapists often focus only on their area of expertise and being supervised by someone from a different profession helped them gain a broader understanding of the patient’s needs:

‘During varsity, I only focussed on the patient’s difficulties instead of looking at them holistically, but now collaborating with other professionals made it easier to see the patient as a whole while giving individualised care.’ (P3, Female, AUD, 23)

### Theme 3: Dissatisfaction with supervision

Most of the participants expressed dissatisfaction with cross-disciplinary supervision, this being indicated in the two sub-themes of the lack of supervision and effect on career advancement.

#### Sub-theme 3.1: The lack of supervision

Some participants indicated that the absence of a supervisor onsite, precisely one in the same discipline, resulted in a lack of guidance and direction during critical tasks, that provision should have been made for them to access suitable persons who worked nearby to address their concerns:

‘I did not feel like I had a supervisor … I had no one to advise and correct me when I was wrong. I feel like I was thrown into the deep end.’ (P1, Female, OT, 24)

‘They should at least allow us to go to other hospitals with experienced therapists so we can learn more … This supervision type is unnecessary; at least they should give us experienced online supervisors.’ (P7, Female, SLT, 24)

#### Sub-theme 3.2: Effects on career advancement

Participants indicated that the limited and inadequate supervision received negatively impacted their career progression at a later stage, as they did not learn any new skills or develop competencies in dealing with new situations. This compromised their clinical development when applying for jobs after CS, as they were unable to demonstrate how they effectively dealt with complex scenarios or learned to manage challenging situations:

‘One-panel member during my post community service interview told me that she can tell when someone did not have a supervisor during their community service … ’ (P4, Female, AUD, 24)

One participant recalled when she was offered a post, she was required to observe several procedures before being allowed to work independently. This was because of her lack of profession-specific skills that should have been acquired during her CS year had she had appropriate supervision:

‘When I started work post community service, I had to observe first for a week … This helped me learn new skills from experienced therapists.’ (P6, Female, SLT, 25)

### Theme 4: Recommendations for effective cross-disciplinary supervision

Two sub-themes emerged regarding recommendations, these being building collaborative relationships and learning effective communication strategies.

#### Sub-theme 4.1: Building collaborative relationships

Participants advised that therapists who might be cross-disciplinary supervised should form collaborative relationships with others in the same profession working elsewhere during their training year. This would enable them to find support from those in similar positions and provide an opportunity for them all to benefit from each other’s challenges and experiences:

‘Having a relationship with therapists from other hospitals helped, because I could ask questions and advice. This made me more effective and confident when delivering services because I felt like I had someone to run to should things become challenging.’ (P5, Female, PT, 24)

‘My communication skills helped me when I asked a speech therapist from a different hospital to assist me with ordering, since my supervisor was unable to assist.’ (P7, Female, SLT, 24)

#### Sub-theme 4.2: Effective communication strategies

Participants mentioned how being cross-disciplinary supervised sharpened their communication skills, improved their ability to effectively communicate with other professionals and ask for assistance when needed:

‘To survive this experience, you must have effective communication strategies, know what you need, and be able to make your point across.’ (P5, Female, PT, 24)

## Discussion

The study aimed to explore the experiences of newly qualified rehabilitation therapists about supervision and support received during the CS year. The results have shed light on the complex interactions of cross-disciplinary supervision and uncovered a range of obstacles and advantages that significantly affected the growth of inexperienced therapists. Despite the emergence of specific benefits, such as improved learning opportunities and comprehensive patient care, it is essential to consider the challenges and dissatisfaction they expressed. This conversation seeks to place these findings in the larger context of professional growth, working across disciplines, the impact on future actions and suggestions for improving the supervision process.

Regarding the challenges associated with cross-disciplinary supervision, the supervisors’ lack of pertinent knowledge was a significant obstacle to successful clinical development and supervision during the participants’ CS year. They were frustrated by their supervisors’ lack of understanding in specialised areas, which affected their ability to deal with complex patient cases and affected their overall development. Participant 6 faced this difficulty, emphasising the lack of efficient communication and direction in crucial moments. Supervisees lacked a shared understanding of specific clinical scenarios, resulting in feelings of isolation and a lack of support, which decreased their confidence in making decisions and managing patients. Kilminster, in an article on clinical supervision, highlighted the need for supervisors to understand different disciplines in order to be able to create a conducive environment for learning and successful supervision.^13^ Crocket et al., in a study on cross professional supervision, concluded that addressing this lack of knowledge is essential to improving patient care quality and providing supervisees with the necessary mentorship for success in their professional positions.^5^

Regarding sub-theme 2, power dynamics in cross-disciplinary supervision can complicate the supervisory relationship, with participants in disciplines other than physiotherapy expressing feelings of being marginalised, especially when advocating for their field. Their perceptions were that certain professions were regarded as being more valued in the hospital and therefore holding more power in a supervisory role, which can lead to neglecting the contributions from less dominant fields. This was common in departments where there were more physiotherapy than audiology staff members, the former having more authority and was reported in a study conducted by Looman et al.^14^ This lack of support may cause new therapists to feel unsure of themselves, as they believe their views are not relevant or respected.

Literature on power dynamics in cross-disciplinary teams indicate that an unequal representation can impede collaboration and reduce the overall efficiency of service provision.^8^ A study in the United Kingdom (UK) looking at the power differentials between supervisors and those they supervised recommended that supervisor transparency regarding their abilities and limitation reduces their power and improves supervision.^15^ Sriram et al.^16^ recommended that supervision systems must prioritise fairness across different fields to promote a more inclusive environment to ensure that all viewpoints are acknowledged and respected. This may include establishing official channels for receiving feedback and support, thereby enabling new therapists to voice their opinions and ideas without worrying about backlash.^16^

As indicated in sub-theme 3, resource constraints are a reality for public sector healthcare workers in SA. However, these limitations further complicated the oversight experience for participants, particularly when the supervisor was expected to motivate for appropriate equipment but was not able to because of competing priorities or a lack of knowledge. These limitations posed a significant obstacle to the supervision and delivery of services. Participants reported struggling to deliver high-quality care because of insufficient tools and materials in their work environments. Studies show that resource limitations in healthcare can cause heightened stress, burnout and adverse patient results.^17^ This issue is particularly problematic in rehabilitation therapy, where the right tools are crucial for successful assessment and treatment. Participants felt frustrated at having to transfer patients to better-equipped facilities, when they believed that with the correct equipment, they could have dealt with the cases themselves. A study done by De Stefano et al. showed that this scenario damages inexperienced therapists’ trust and hinders their opportunities for practical learning and skill enhancement.^15^

There were however some advantages of cross-disciplinary supervision during participants’ years of CS, with increased learning opportunities highlighted by participants, as indicated in sub-theme 1. Several participants found that the lack of a supervisor within their field inspired them to advance their professional growth proactively. Their reflection showed how they were expected to do research and keep up with current practices while navigating clinical situations without direct supervision. Such instances have shown how interdisciplinary environments can promote autonomous learning and perseverance, helping trainees develop into more adept and informed professionals.^18^ This situation can result in better patient care as people use their newly acquired knowledge and abilities in the field, indicating that obstacles can effectively drive personal and professional advancement.^19^

Sub-theme 2 highlighted how cross-disciplinary supervision is recognised as a powerful influence in advancing individuals’ critical administrative and management competencies. While the clinical learning setting may have deficiencies, the varied administrative skills obtained can be highly beneficial in navigating healthcare system complexities. Rehabilitation therapists need to learn how to competently undertake administrative tasks while juggling clinical duties and organisational needs. They observed that their time in cross-disciplinary environments gave them abilities and competencies that would benefit future positions. This proactive experience enhanced their comprehension of management processes and created a feeling of pride and achievement in their learning journey, despite facing a number of challenges.^20^ Participant 3 mentioned that being supervised by someone from a different field made her consider things beyond her usual frame of reference and illuminates how diverse viewpoints can lead to creative problem-solving approaches. In addition, participant 5 pointed out the importance of mediating conflicts within a team, underscoring the practical skills acquired by witnessing various management techniques essential for successful team interactions. These reports highlight how supervision across different disciplines helps junior staff to acquire crucial skills to address obstacles and prepare for future leadership positions in varied surroundings.

Participants found an increase in their comprehension of holistic patient care because of cross-disciplinary supervision, as outlined in sub-theme 3, and their collaboration with experts from diverse fields helping them broaden their perspectives beyond their areas of specialisation. Participants emphasised that working with other professionals changed their focus from only dealing with a patient’s problems in their field to seeing them as complete individuals with various needs. Huljev highlights that in healthcare, it is essential to have a comprehensive understanding of the connection between the physical, emotional and social factors that impact a patient’s health to provide effective treatment.^21^ The lessons learned from these cross-disciplinary experiences show the significance of interprofessional education and practice, improving the skills of individual practitioners, resulting in more thorough and tailored patient care.^5^

Participants displayed widespread dissatisfaction with the lack of supervision across different disciplines, as noticed in sub-theme 1. Numerous individuals conveyed emotions of being left behind and not being good enough, as expressed by one participant who felt that she was ‘thrown into the deep end’ without proper assistance or backing. Moreover, they requested access to seasoned therapists in nearby hospitals in order to obtain additional hands-on learning experiences in an effort to improve their skills and boost their confidence. The expressed dissatisfaction emphasises the importance of organised and encouraging supervisory systems that focus on mentorship and offer interaction with seasoned professionals. A study conducted in the UK by Kilminster regarding adequate educational and clinical supervision states that the lack of correct supervisory support can lead trainees or novice therapists to have difficulties dealing with complicated clinical scenarios, thereby negatively affecting their development and the level of care they deliver.^22^

As indicated in sub-theme 2, the participants felt that their cross-disciplinary supervision experience negatively affected their career progression, as the insufficient support could impede their professional growth. Several individuals were worried that the insufficient oversight during their CS year resulted in apparent deficiencies in their abilities, this being evident during job interviews and when starting their first placement. For example, a participant (P4) mentioned how an interviewer noticed inadequate mentorship, which affected her perceived preparedness for the position. Another participant (P6) mentioned that she had to dedicate a week to observing procedures when she began her job, as her CS experience had not provided enough practical experience. A study conducted in the United States (US) in 2018 identified that counsellors who were supervised by a different cadre felt ambivalent about their competencies in independent practice.^23^ Appropriate CS supervision is essential for immediate job preparedness and long-term professional accomplishments.^24^

Building collaborative relationships across disciplines can be fostered by cross professional supervision, improved therapists’ understanding of holistic patient care, promoting creativity and effectively tackling complex problems, as indicated in sub-theme 2. Cross-disciplinary collaboration has been shown to be essential in rehabilitation treatment as it enables therapists to integrate information from other disciplines and provide more comprehensive care to patients.^25^ A systematic review conducted in 2017, using data from high income countries such as the US and the UK, showed that interprofessional collaboration improved patient outcomes, as therapists better understand the needs of their patients and encouraged comprehensive inventive treatment strategies.^18^ Inexperienced therapists can enhance their professional efficacy by implementing a complete plan for understanding patient care.^26^ Suggestions for enhancing successful collaboration involve promoting communication, setting defined objectives and fostering the exchange of knowledge.^27^ By implementing these tactics, novice therapists can establish productive collaborations that result in positive results in various areas of patient care.^27^

As noticed in sub-theme 2, the participants emphasised that mastering the challenges of cross-disciplinary supervision required acquiring proficient communication skills, enabling them to express their needs and work efficiently with colleagues from diverse backgrounds. Effective communication tactics in cross-disciplinary environments have been shown to be essential for improving the supervisory process and professional growth.^28,29^ According to the participants, effectively communicating ideas and asking for help was important for succeeding in this challenging situation.

Participant 7 shared an experience about how communication was essential for navigating obstacles in professional settings. This focus on communication made it easier for team members to interact and encouraged a culture of collaboration and support. Participants who improved these skills reported increased confidence when interacting with other professionals, resulting in better teamwork and patient care results.^30^ Introducing specific training programmes to improve communication strategies in interdisciplinary supervision can therefore significantly help new professionals. These efforts would give them the necessary skills to navigate various professional environments, resulting in improved teamwork and enhanced educational opportunities.

## Limitations

A number of limitations may have affected the findings, including the small sample size, participants were all female and only one was based in an urban area. As it was only conducted in KZN, the findings may not be relevant to other provinces in the country. Another limitation of this study was the research setting and recruitment method. Relying on social media for participant recruitment may have excluded CS therapists who either lacked access to social media platforms or chose not to engage with them for research purposes. In addition, the specific social media platforms used for recruitment may not have been comprehensive enough to reach the entire population of CS therapists, potentially resulting in a sample that is not fully representative of all therapists in KZN. This could limit the diversity of perspectives and experiences captured in the study.

## Conclusion

Examining supervision across rehabilitation disciplines uncovered a complex terrain that can both benefit and negatively affect novice therapists’ training and experiences. Although there were advantages, such as improved learning opportunities and comprehensive patient care, difficulties presented by communication obstacles, limited resources and power dynamics need to be addressed. Both provide opportunities to strengthen the measures that need to be developed and implemented to enable CS therapists to optimise their year of services, learn from the experience and provide quality services.

Healthcare institutions should empower new therapists by investing in training for supervisors, fostering collaborative partnerships, improving communication skills, addressing resource limitations and setting up effective feedback systems. Promoting an inclusive and well-equipped supervisory experience will ultimately boost therapists’ professional effectiveness and enhance patients’ results. By reevaluating the supervisory measures that are available on a regular basis, including the opinions of CS rehabilitation therapists, progress can be made to address the challenges that are inevitable in resource constrained settings. This will nurture a cadre of young professionals who are equipped not only to provide quality services but also to work in multidisciplinary teams to provide holistic patient care.
